# The Impact of COVID-19 on Long-Term Mortality in Maintenance Hemodialysis: 5 Years Retrospective Cohort Study

**DOI:** 10.3390/jcm14197081

**Published:** 2025-10-07

**Authors:** Ioana Adela Ratiu, Lorena Filip, Corina Moisa, Cristian Adrian Ratiu, Nicu Olariu, Iulia Dana Grosu, Gabriel Cristian Bako, Andrei Ratiu, Mirela Indries, Simona Fratila, Danut Dejeu, Gianina Adela Gabor, Luciana Marc

**Affiliations:** 1 Faculty of Medicine and Pharmacy, University of Oradea, 1st December Square 10, 410073 Oradea, Romania; ioana.ratiu@didactic.uoradea.ro (I.A.R.); bako_gabriel@uoradea.ro (G.C.B.); mirela.indries@didactic.uoradea.ro (M.I.); sfratila@uoradea.ro (S.F.); ddejeu@uoradea.ro (D.D.); 2 Nephrology Department, Emergency Clinical Hospital Bihor County, 12 Corneliu Coposu Street, 410073 Oradea, Romania; 3 Faculty of Pharmacy, University of Medicine and Pharmacy “Iuliu Hatieganu” Cluj-Napoca, Victor Babeș Street 8, 400012 Cluj-Napoca, Romania; lfilip@umfcluj.ro; 4 Academy of Romanian Scientists (AOSR), 3 Ilfov Street, 050044 Bucharest, Romania; 5 Dentistry Department, Faculty of Medicine and Pharmacy, University of Oradea, 1st December Square 10, 410073 Oradea, Romania; 6 Department of Internal Medicine II—Division of Nephrology, “Victor Babeș” University of Medicine and Pharmacy, 300041 Timisoara, Romania; olariu.nicu@umft.ro (N.O.); grosu.iulia@umft.ro (I.D.G.); marc.luciana@umft.ro (L.M.); 7 Center for Molecular Research in Nephrology and Vascular Disease, Faculty of Medicine, “Victor Babeș” University of Medicine and Pharmacy, 300041 Timisoara, Romania; 8 Faculty of Dentistry, University of Medicine and Pharmacy “Iuliu Hatieganu” Cluj-Napoca, Victor Babeș 8, 400347 Cluj-Napoca, Romania; ratiu.andrei@elearn.umfcluj.ro; 9 Infectious Diseases Department, Emergency Clinical Hospital, 410167 Oradea, Romania; 10 Faculty of Electrical Engineering and Information Technology, Computers and Information Technology Department, University of Oradea, Universitatii Street 1, 410087 Oradea, Romania; gianina@uoradea.ro

**Keywords:** COVID-19 infection, hemodialysis, anti-SARS-CoV2 vaccination, mortality, cardiac mortality

## Abstract

**Background**: Hemodialysis (HD) patients are a highly vulnerable population with elevated mortality driven by comorbidities and dialysis-specific factors. While most studies focused on intra-pandemic outcomes, long-term effects remain underexplored. We aimed to evaluate 5-year mortality and the impact of COVID-19 vaccination in chronic HD patients. **Methods**: A retrospective study was conducted on 211 HD patients monitored between 2020 and 2024. Outcomes included overall and cardiovascular mortality, risk factors in COVID-19-positive patients, and vaccination impact. Logistic regression identified independent predictors. **Results**: The cohort had a mean age of 65.6 ± 13.3 years, with 55.9% males and mean dialysis vintage of 6.9 ± 5.5 years. Overall mortality reached 53.6%, while 38.4% were vaccinated. Predictors of all-cause mortality included age (OR = 1.078, *p* < 0.001), BMI (OR = 0.868, *p* < 0.001), hemoglobin (OR = 0.581, *p* < 0.001), phosphorus (OR = 1.351, *p* = 0.025), dialysis adequacy (OR = 0.138, *p* = 0.013), and ischemic cardiopathy (OR = 0.327, *p* = 0.009). In COVID-19-positive patients, mortality was associated with age (OR = 1.069, *p* = 0.002), low hemoglobin (OR = 0.642, *p* = 0.014), BMI (OR = 0.885, *p* = 0.009), CRP (OR = 1.015, *p* < 0.001), and coronary artery disease (OR = 5.68, *p* < 0.001). Cardiovascular disease was the leading cause of death (44.6% in COVID-19-positive vs. 73.3% in negatives, *p* = 0.006). Vaccination significantly reduced COVID-19-related mortality (OR = 0.023, *p* = 0.005) but did not influence overall or non-COVID mortality. **Conclusions**: Five-year mortality in HD patients remained high, mainly cardiovascular, and was strongly influenced by age, BMI, hemoglobin, dialysis adequacy, and comorbidities. COVID-19 vaccination substantially reduced COVID-related mortality but did not alter all-cause outcomes. These findings support vaccination and careful risk stratification in HD populations for future pandemics.

## 1. Introduction

Chronic kidney disease (CKD) constitutes a significant global public health concern, with a substantial impact on both mortality and morbidity [1]. By 2040, CKD is anticipated to emerge as the sixth predominant cause of mortality globally [2]. As glomerular filtration rate (GFR) declines and albuminuria increases, the risk of death from cardiovascular, infectious, and metabolic complications rises, reducing the likelihood that patients will initiate renal replacement therapy (RRT). Hemodialysis (HD) remains the most used form of RRT, far surpassing kidney transplantation and peritoneal dialysis [3].

**Figure 1:**
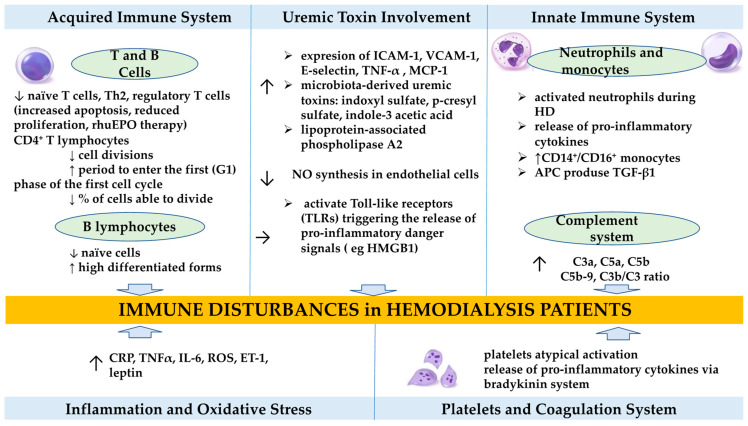
The imbalance between innate and adaptive immunity. Legend: rhuEPO-recombinant human erythropoietin, CRP-C-reactive protein, TNF-tumor necrosis factor, IL-interleukin, ROS-reactive oxygen species, ET-endothelin, ICAM-intercellular adhesion molecule, VCAM-vascular cell adhesion molecule, MCP-monocyte chemoattractant protein, HMGB-high mobility group box proteins, APC-antigen presenting cells.

Patients undergoing HD exhibit both innate and adaptive immune dysfunction, as shown in both experimental and vivo studies [4,5]. This dysfunction is driven by uremic toxins, dialysis techniques, malnutrition, and chronic inflammation (Figure 1). An exaggerated immune reaction to specific stimuli, combined with impaired clearance of proinflammatory cytokines and the proliferation of CD4+CD28− and CD14+CD16++ T-cells, has been associated with increased risks of cardiovascular, neo-plastic, and infectious diseases [6,7,8,9]. These patients also present elevated levels of proinflammatory cytokines, including IL-1β, TNF-α, and IL-6, as well as increased leptin and cystatin C concentrations [10,11,12]. On the other hand, the imbalance between innate and adaptive immunity, feature of CKD, contributes to a diminished vaccine response.

The recent COVID-19 pandemic had devastating effects on patients undergoing hemodialysis [13]. The risk resulted not only from the direct effects of infection with severe acute respiratory syndrome coronavirus 2 (SARS-CoV-2), but also to the limited access to medical evaluation and treatment during the pandemic [14]. Preventive measures such as patient isolation were largely ineffective, given the continuous intercommunity movement, the need for regular transport to dialysis centers, and the underdiagnosis of infection due to reliance on rapid screening tests in HD units. In addition, post-COVID syndrome (long COVID), due to its chronic multi-organ involvement—predominantly affecting the cardiopulmonary system—had a decisive impact on the long-term mortality of patients undergoing hemodialysis [15]. Immune dysregulation in ESRD patients contributed to the development of severe disease phenotypes, including chronic respiratory failure, prolonged need for oxygen therapy, and increased susceptibility to cardiovascular, infectious, and neo-plastic comorbidities [16].

SARS-CoV-2 enters host cells and bind to the angiotensin-converting enzyme 2 (ACE2) receptor, a process facilitated by transmembrane protease serine 2 (TMPRSS2). This mechanism explains the predominant viral replication in organs with high ACE2 ex-pression, such as the lungs, kidneys, and heart and raised concern regarding the use of angiotensin-converting enzyme inhibitors (ACEi) and angiotensin receptor blockers (ARBs) during the pandemic.

COVID-19 vaccination became available in 2020 and included mRNA-based vaccines, non-replicating viral vector vaccines, and inactivated virus vaccines. In our country, the primary vaccines administered were BNT162b2/Pfizer, mRNA-1273/Moderna, AstraZeneca, and Johnson & Johnson. The effectiveness of vaccination in patients with CKD—particularly those with ESRD on HD—has been evaluated in both clinical and laboratory studies [17,18]. Given the immunological alterations in this population, suboptimal anti-spike antibody titters were anticipated. However, despite diminished humoral responses, cellular immunity in HD patients was sufficient to provide protection against severe forms of COVID-19 [19]. Overall, evidence supports that complete vaccination regimens significantly reduce morbidity and mortality in this vulnerable population, despite initial concerns regarding efficacy, autoimmunity, or adverse reactions.

Considering the number of HD patients under our care and the fact that our hospital was designated as a regional COVID-19 treatment center during the pandemic, we aimed to evaluate the impact of SARS-CoV-2 infection on long-term morbidity and mortality in the HD population. We believe that our findings will be of benefit not only for the patients—through the identification of infection-associated risk factors—but also to healthcare providers, by offering data that may inform the development of treatment protocols in future pandemic scenarios.

## 2. Materials and Methods

We conducted a retrospective cohort study over a 5-year period (1 January 2020–31 December 2024) aimed to evaluate the impact of SARS-CoV-2 infection on the long-term outcomes of this fragile patient population (HD patients), with a particular focus on mortality during, and especially after, the pandemic.

The study was carried out in one of the largest tertiary hospitals in Western Romania and received approval from the Ethics Committee of the Bihor County Emergency Clinical Hospital (approval no. 2376/23.01.2025). All patients included in the study had signed informed consent at the time of hospitalization. Data were collected from GP file (2020–2024) and specialized outpatient clinic documentation.

### 2.1. Study Objectives

1. To evaluate all-cause mortality during the follow-up;
2. To assess COVID-19–related and cardiovascular mortality;
3. To analyze the effects of COVID-19 vaccination on mortality in hemodialysis patients.

### 2.2. Data Collection

Inclusion criteria: (a) Patients receiving chronic hemodialysis in 2020; (b) age over 18 years.

Exclusion criteria: Patients who initiated chronic hemodialysis after 2020.

A total of 211 patients from County Emergency Clinical Hospital Oradea were included in the study and followed between 2020 and 2024. The mean follow-up period was 3.6 ± 1.6 years. The patients who passed at least one time during the follow-up through COVID-19 infection were included in the COVID-19-positive group [COVID (+)] and the rest were in COVID-19-negative group [COVID (-)]. We identified 149 patients with COVID (+) and 62 patients COVID (-). A total of 81 patients received SARS-CoV-2 vaccination, the vast majority with mRNA-based vaccines. COVID-19 vaccination status (yes/no) was considered positive if the patient had received at least two vaccine doses.

We monitored the incidence of COVID-19 infection over the 5-year study period, during which features of long COVID may also manifest, and recorded mortality events along with associated risk factors. For this purpose, multivariate analyses were performed on demographic variables, laboratory parameters, and comorbid conditions commonly associated with this patient population.

The demographic parameters evaluated were age, dialysis vintage, and sex, while biometric parameters included body mass index (BMI) and body surface area (BSA). The statistical analysis also incorporated the underlying renal disease leading to kidney failure, namely chronic glomerulonephritis (CGN), vascular nephropathy (VN), diabetic nephropathy (DN), and autosomal dominant polycystic kidney disease (PKD). We additionally considered a history of prior transplantation and the presence of comorbidities such as diabetes mellitus, arterial hypertension, and malignancies. Demographic data were collected from patients’ GP files and outpatient clinic documentation. Informed consent for the collection and use of personal medical data was obtained from all patients, either at the time of hospital admission or during specialist outpatient visits.

We compared hemodialysis-related characteristics, including the presence of an arteriovenous fistula (AVF) and Kt/V (K = dialyzer clearance, t = dialysis time, V = volume of urea distribution) as an indicator of dialysis adequacy. Laboratory work-up included hemoglobin, C-reactive protein, serum creatinine, calcium, phosphorus, intact parathyroid hormone (iPTH), albumin, and total serum cholesterol as markers of nutritional status, as well as serum bicarbonate.

The following methods and analyzers were used for laboratory evaluations: (a) Hb measurement using impedance on the UNICEL DxH 900, Beckman Coulter, Danvers MA, USA hematology analyzer; (b) for the measurement of hsC-reactive protein (hsCRP), serum albumin, total cholesterol (T-Chol), calcium, phosphate, iPTH and bicarbonate, a turbidimetric method was used with the B04078-AU5811, Beckman Coulter, Danvers MA, USA chemistry analyzer.

The diagnosis of COVID-19 was established by rapid antigen testing and confirmed through the detection of SARS-CoV-2 RNA in nasopharyngeal swab samples using real-time polymerase chain reaction (RT-PCR). The assay had a detection limit of cycle threshold (Ct) 40 and was carried out using the NIMBUS_CFX96 analyzer. Testing was performed in symptomatic patients or in those with known COVID-19 exposure.

Statistical analysis was conducted using Jamovi 2.7.2.0, and confirmed using Stata/SE 17.0 for Windows (64-bit×86-64) Revision 21.05.2024, copyright 1985–2021 4905 Lakeway Drive, College Station, Texas 77845-4512, USA (state license serial number 401809208832). We assessed continuous variables using *t*-tests, while categorical variables were analyzed with the Pearson Chi-square test (χ^2^). Continuous variables were reported as means with their respective standard deviations (SDs). Logistic regression was employed to assess the factors associated with mortality and their relative impact.

## 3. Results

### 3.1. Baseline Characteristics of the Study Cohort; Comparative Evaluation of COVID-19-Positive and -Negative Patients

The mean age of the patients included in our study was 65.659 years, with a slight predominance of male gender, and a mean dialysis vintage of 6.92 years. An AVF was used as the vascular access for HD in 65.87% of patients, while the remainder had a central venous catheter. A history of kidney transplantation was present in 8.05% of patients. Arterial hypertension was found in 90.09% of patients, whereas diabetes mellitus (DM) was present in 31.27%. Neoplastic disease was identified in 15.63% of patients. The underlying renal disease was most commonly CGN. The mean BMI placed the study group in the overweight category. Laboratory data showed that patients were within the therapeutic target for serum hemoglobin (10.7 ± 1.50 g/dL) and had adequate dialysis efficiency (Kt/V 1.57 ± 0.314), without major calcium–phosphate imbalances, although iPTH levels were moderately elevated (373.29 ± 425.68 pg/mL). Nutritional status was adequate, as reflected by a mean serum albumin of 3.73 ± 0.48 g/dL and total cholesterol of 152.47 ± 39.39 mg/dL. COVID-19 infection was diagnosed in 70.61% of patients, while 38.38% of the cohort had received vaccination. The overall mortality was 53.55%, with a mean age at death of 69.51 ± 11.943 years.

When comparing patients with COVID-19 infection to those without, the COVID-19-positive group was significantly older (66.86 ± 11.801 vs. 62.774 ± 16.092, *p* = 0.021) and had a higher body mass index (26.52 ± 5.949 vs. 24.32 ± 6.462, *p* = 0.009), with 61.4% being overweight or having grade I–II obesity. Morbid obesity was present in 5 patients (3.3%). No statistically significant differences were observed between the two groups regarding age at death, gender, dialysis vintage, history of transplantation, diabetes mellitus, or arterial hypertension.

Regarding the underlying cause of CKD, vascular nephropathy was significantly more frequent among COVID-19-positive patients (*p* = 0.043), whereas CGN was more commonly observed in the COVID-19-negative group (*p* = 0.027). Anti-COVID-19 vaccination was significantly less frequent among patients who developed COVID-19 compared with those who did not (34.89% vs. 49.77%, *p* = 0.008). Laboratory data did not reveal any statistically significant differences between the two groups. All data are presented in Table 1.

When analyzing overall mortality, 113 (53.55%) patients died during the follow-up period. The proportion of deaths within each group was comparable, with 55.7% in COVID-19-positive patients and 48.39% in COVID-19-negative patients (*p* = 0.412).

In COVID-19-positive patients, COVID-19 infection itself accounted for 22.81% of patients (40.96% of deaths, respectively) making it the second leading cause of mortality in this group. However, cardiovascular disease was the primary cause of death in both groups. Among COVID-19-negative patients, cardiovascular causes represented a significantly higher proportion of all deaths compared with the COVID-19-positive group (73.33% vs. 44.57% of all deaths from each group, *p* = 0.006).

Non-COVID infections accounted for 4.81% of deaths in the COVID-19-positive group (2.68% of COVID-positive patients) and 10% in the COVID-19-negative group (4.83% of COVID-negative patients), with no statistically significant difference (*p* = 0.570). Deaths due to malignancy represented 8.43% of causes in the COVID-19-positive group and 10% in the COVID-19-negative group (4.69% vs. 4.83% of the patient’s groups, *p* = 0.907).

### 3.2. Evaluation of Mortality

#### 3.2.1. Factors Influencing Overall Mortality

By linear regression, only a few factors were corelated with higher mortality (Table 2). In the study cohort, deceased patients were significantly older compared to survivors (69.51 ± 11.94 vs. 61.21 ± 13.45 years; *p* = 0.00001), while dialysis vintage was similar between the two groups (6.99 ± 6.01 vs. 6.85 ± 4.84 years; *p* = 0.424). Paradoxically, patients with a higher BMI—above the mean value of 26.69—demonstrated better survival. To account the potential confounding factors in the analysis of the relationship between BMI and mortality, we conducted a subgroup analysis by stratifying patients into two categories: those with a BMI above and below 25.19 (mean BMI in deceased patients). Patients with a BMI above 25.19 were observed to be younger (mean age 65.25 ± 10.63 vs. 66.02 ± 15.34 years, *p* = 0.673), had a shorter duration on hemodialysis (6.32 ± 4.103 vs. 7.46 ± 6.441 years, *p* = 0.135), higher hemoglobin levels (10.726 ± 1.6 vs. 10.680 ± 1.4, *p* = 0.827), higher serum albumin concentrations (3.858 ± 0.367 vs. 3.625 ± 0.541, *p* < 0.001), and lower parathyroid hormone (PTH) levels (350.38 ± 353 vs. 394.6 ± 478, *p* = 0.465). However, this group also exhibited a lower Kt/V (1.517 ± 0.28 vs. 1.624 ± 0.337, *p* = 0.015) and higher serum phosphorus levels (5.272 ± 1.64 vs. 4.918 ± 2, *p* = 0.171). No statistically significant differences were observed regarding gender, prior kidney transplantation, or type of vascular access.

Diabetes mellitus was more frequent among patients who died, although without statistical significance (39.82% vs. 21.42%; *p* = 0.066). Both deceased and surviving patients were, on average, overweight; however, those who died had a significantly lower body mass index (25.19 ± 6.57 vs. 26.69 ± 6.60 kg/m^2^; *p* = 0.04).

Regarding laboratory findings, deceased patients were significantly more anemic, with lower hemoglobin levels compared to survivors (10.25 ± 1.57 vs. 11.16 ± 1.20 g/dL; *p* = 0.00002), had reduced dialysis efficiency, and demonstrated poorer nutritional status, as reflected by lower serum albumin (3.65 ± 0.566 vs. 3.83 ± 0.332 g/dL; *p* = 0.0036) and serum creatinine levels (7.22 ± 2.40 vs. 9.67 ± 15.05 mg/dL; *p* = 0.045). Administration of antiviral therapy against COVID-19 did not significantly influence mortality (*p* = 0.532). Lopinavir/ritonavir combination was the most frequently used in hospitalized COVID patients, followed by favipiravir and remdesivir. In contrast, the rate of SARS-CoV-2 immunization was significantly lower among deceased patients compared to survivors (29.2% vs. 48.97%; *p* = 0.0032).

Logistic regression analysis revealed that five-year post-pandemic all-cause mortality was significantly influenced by age (OR = 1.078, *p* < 0.001, 95% CI: 1.038–1.119), body mass index (OR = 0.868, *p* < 0.001, 95% CI: 0.803–0.939), hemoglobin level (OR = 0.581, *p* < 0.001, 95% CI: 0.427–0.791), phosphorus (OR = 1.351, *p* < 0.025, 95% CI: 1.039–1.758), and dialysis adequacy (Kt/V, OR = 0.138, *p* = 0.013, 95% CI: 0.029–0.658). However, the CI obtained for the last two parameters is large, suggesting the need for a larger patient sample. CRP is a very reliable mortality predictor, with OR = 1.014, *p* < 0.001, 95% CI: 1.006–1.023). Ischemic cardiopathy proved to be a good mortality predictor (OR = 3.058, *p* 0.009, 95% CI: 1.317–7.101). Neither diabetes mellitus and neoplastic diseases, nor anti-COVID vaccination/treatment demonstrate their predictability regarding general mortality (Table 3).

**Figure 2:**
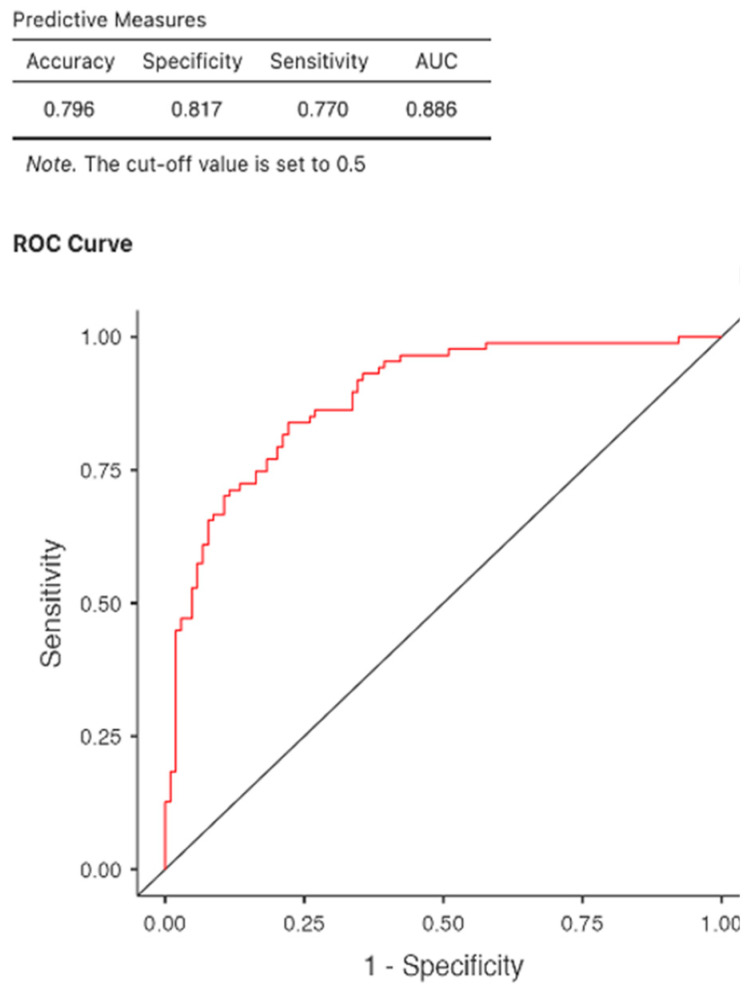
Multiple logistic regression evaluating overall mortality.

The ROC curve analysis yielded an AUC of 0.886 with the best AIC = 190 for this model, indicating high predictive accuracy with good discrimination between patients who died and those who remained alive at the end of the study (Figure 2).

#### 3.2.2. Mortality in COVID-19-Positive Patients

##### Mortality in COVID-19-Positive Versus COVID-19-Negative Patients

When comparing general mortality and cardiovascular mortality between patients with COVID-19 infection and those without SARS-CoV-2, no statistically significant differences were observed with respect to demographic characteristics, associated comorbidities, dialysis-related parameters, or laboratory findings (Table 4).

Cardiovascular disease was the leading cause of death in both groups. Among post-COVID patients, 47.57% of deaths were attributed to cardiovascular causes, compared to 73.33% in COVID-negative patients (*p* = 0.06). No statistically significant differences were observed between the two groups regarding age at death, dialysis vintage, vascular access, comorbidities, or major laboratory parameters.

COVID-negative patients exhibited poorer nutritional status, with a lower body mass index and significantly reduced serum albumin levels compared to post-COVID patients (3.6 ± 0.415 vs. 3.8 ± 0.429 g/dL; *p* = 0.049). The proportion of patients vaccinated against SARS-CoV-2 was lower in the COVID-negative group, with the difference approaching statistical significance (*p* = 0.062).

##### Predictors of Mortality in COVID-19-Positive Patients

Since no significant differences were found between patients with or without COVID-19 infection, we identified the same parameters correlating with mortality in COVID-19-positive patients as those observed for overall mortality: age, diabetes mellitus, BMI, hemoglobin, albumin, calcium, creatinine, total cholesterol, and KTV. Thus, mortality occurred at significantly older ages compared with survivors (70.27 ± 9.98 vs. 62.58 ± 12.57 years, *p* = 0.00002). No significant differences were observed with respect to gender, dialysis vintage, type of vascular access, history of prior transplantation, or presence of arterial hypertension. However, diabetes mellitus was substantially more common among deceased patients than among survivors (39.75% vs. 22.72%, *p* = 0.042).

Regarding the CKD cause, vascular nephropathy predominated among deceased patients, whereas CGN was more common among survivors. Both overweight, surviving patients had a significantly higher BMI compared with those who died, although both groups were in the overweight range (27.87 ± 5.19 vs. 25.45 ± 6.32, *p* = 0.006). (Table 5).

In COVID-19-positive patients, non-survivors exhibited significantly lower levels of hemoglobin (10.32 ± 1.59 vs. 11.13 ± 1.30 g/dL, *p* = 0.0005), total cholesterol (146.54 ± 37.67 vs. 160.86 ± 40.86 mg/dL, *p* = 0.014), and albumin, as an indicator of nutritional status (3.66± 0.61 vs. 3.87 ± 0.36 g/dL, *p* = 0.020), despite a significantly higher Kt/V (1.62 ± 0.25 vs. 1.53 ± 0.35, *p* = 0.040). This can be explained by the lack of medical services that covered all the health issues occurring during the pandemic and immediately after. Dialysis vintage is associated with lower COVID mortality. This apparently paradoxical finding can be explained by the evolving profile of patients starting HD in recent years, who tend to be older and with a high burden of comorbidities. Although vaccination was more frequent among COVID-19 survivors compared with those who died (42.42% vs. 28.91%), this difference did not reach statistical significance.

**Figure 3:**
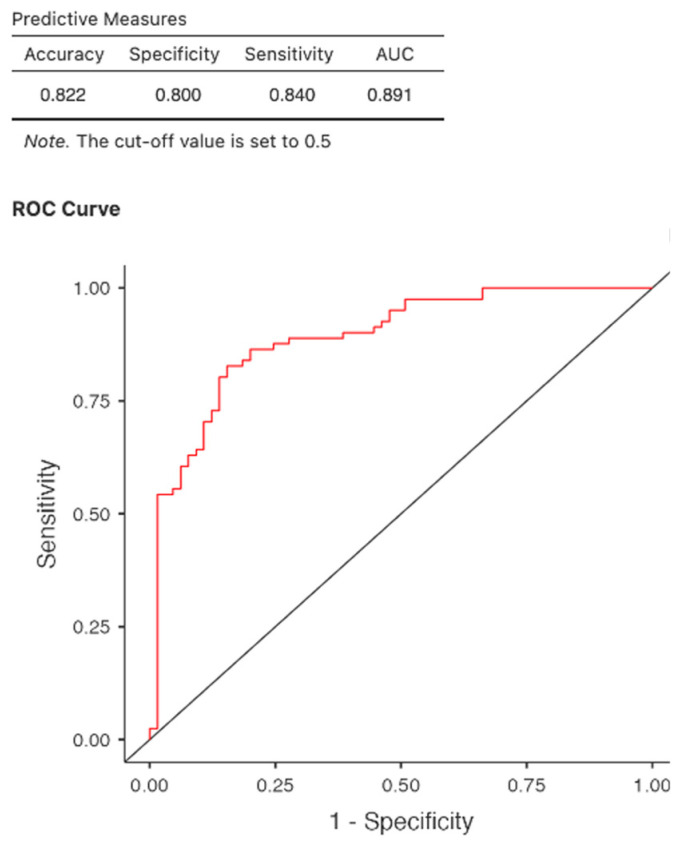
ROC curve. Predictors of mortality in COVID-19-positive patients.

Logistic regression analysis confirmed the predictive value for mortality in COVID-19-positive patients: age (OR = 1.069, *p* 0.002, 95% CI 1.025–1.116), body mass index (OR = 0.885, *p* = 0.009 95% CI 0.808–0.970), hemoglobin (OR = 0.642, *p* = 0.014, 95% CI 0.449–0.915), and CRP (OR = 1.015, *p* < 0.001 95% CI 1.006–1.024). Furthermore, pre-existing coronary artery disease is a significant predictor of death (OR = 5.68, *p* = <0.001, 95% CI 2.16–14.95); however, a larger cohort would be necessary to confirm the result. Neither the presence of diabetes mellitus nor SARS COV2 immunization or treatment impact mortality at patients who passed through COVID-19 infection (Table 6, Figure 3).

The regression model was evaluated using a receiver operating characteristic (ROC) curve. At a cut-off of 0.5, the area under the curve (AUC) was 0.891 with the lowest AIC of 147, indicating very good predictive performance of the model.

##### Predictors of Mortality Due to COVID-19

Linear regression revealed that COVID-19-related mortality was significantly associated with age, dialysis vintage, reduced hemoglobin, albumin and Kt/V levels, and elevated phosphate and CRP, whereas vaccination provided a strong protective effect (*p* < 0.0001). (Table 7).

Logistic regression narrows down the predictors for COVID-19 mortality to hemodialysis vintage (OR = 0.854, *p* 0.013, 95% CI 0.753–0.963), phosphate level (OR = 1.341, *p* 0.044, 95% CI 1.007–1.784), and CRP high levels (OR = 1.012, *p* < 0.001, 95% CI 1.006–1.019). Surprisingly, each additional year on HD was associated with about a 15% reduction in the odds of COVID-19 death.

**Figure 4:**
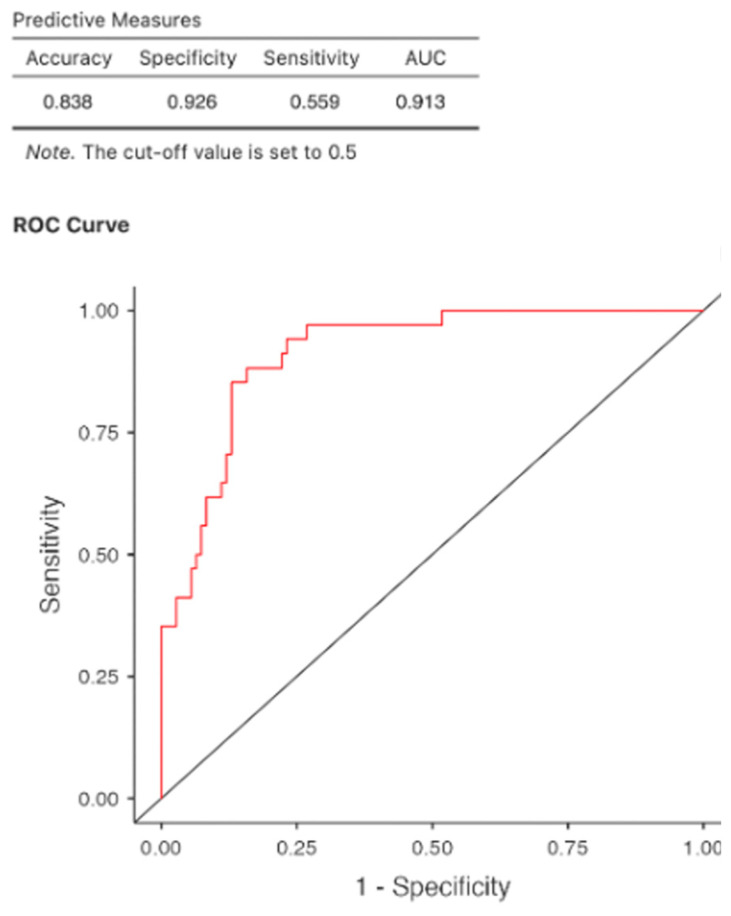
ROC curve. Multiple logistic regression- COVID-19-related mortality.

Anti-SARS-CoV2 immunization (OR = 0.041, *p* 0.003, 95% CI 0.05–0.338) significantly reduces the COVID-19 induced mortality. Adjusted logistic regression analysis indicated that COVID-19 vaccination was associated with a ~96% reduction in the odds of mortality compared with unvaccinated patients (OR = 0.041, *p* = 0.003). However, the wide 95%CI suggests uncertainty in the exact size of effect, possibly due to the small sample size (Table 8, Figure 4).

The ROC curve analysis for our model yielded an AUC of 0.913 with the lowest AIC, indicating a very high predictive performance of the logistic model.

#### 3.2.3. Cardiovascular Disease-Related Mortality

Cardiovascular mortality was associated with older age (70.72 ± 11.76 vs. 61.21 ± 13.451, *p* = 0.0002), with longer history of hemodialysis (8.79 ± 6.69 vs. 6.85 ± 4.836, *p* = 0.0001), with higher hs-CRP reflecting the inflammatory status (54.136 ± 61.764 vs. 29.790 ± 47.254, *p* = 0.007), and with a high frequency of diabetes mellitus (*p* = 0.048) compared to the survivors. They have a lower hemoglobin level (10.43 ± 1.55 vs. 11.16 ± 1.195, *p* = 0.0007), a lower kt/v (1.53 ± 0.33 vs. 1.63 ± 0.264, *p* = 0.023), and a lower BMI (24.35 ± 6.77 vs. 26.69 ± 6.602, *p* = 0.013) (Table 9).

Logistic regression analysis (AUC = 0.828) showed that cardiovascular mortality was modestly influenced by older age (OR = 1.061, *p* = 0.010, 95% CI: 1.014–1.115) and dialysis vintage (OR = 1.082, *p* = 0.064, 95% CI: 0.995–1.180). Cardiovascular mortality was inversely associated with body mass index (OR = 0.818, *p* < 0.001; 95% CI: 0.729–0.916) and serum hemoglobin levels (OR = 0.466, *p* = 0.001; 95% CI: 0.300–0.724). The presence of pre-existing cardiovascular disease was a strong risk factor for cardiovascular death (OR = 5.837, *p* < 0.001, 95% CI: 2.053–16.594).

**Figure 5:**
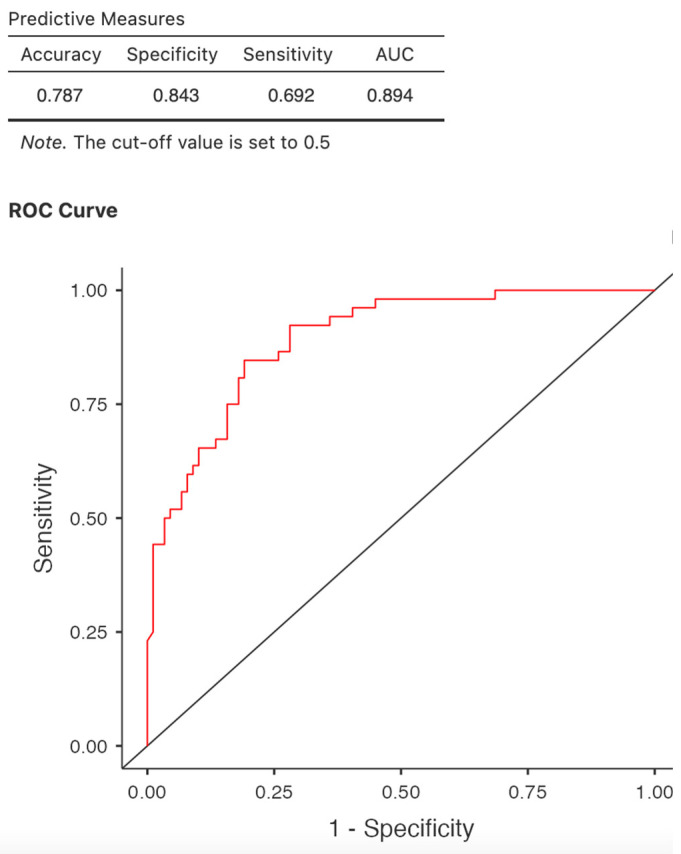
ROC curve. Multivariable logistic regression analysis of predictors of cardiovascular mortality.

Cardiovascular mortality was not associated with vaccination status, history of COVID-19 infection, or parameters of mineral-bone metabolism. Although diabetes mellitus approximately doubled the risk of cardiovascular death (OR = 2.609, *p* = 0.08, 95% CI: 0.866–7.857), this did not reach statistical significance, suggesting the need for a larger cohort to confirm its impact (Table 10, Figure 5).

### 3.3. Impact of Vaccination on Outcomes of Hemodialysis Patients

Demographic characteristics were similar between SARS-CoV-2-immunized and non-immunized patients (age: 66.37 vs. 67.22 years, *p* = 0.270; dialysis vintage: 6.96 ± 5.29 vs. 6.90 ± 5.61 years, *p* = 0.468). No statistically significant differences were observed regarding comorbidities, primary kidney disease, vascular access for hemodialysis, or laboratory parameters. However, patients who declined vaccination had lower dialysis adequacy (Kt/V: 1.53 ± 0.322 vs. 1.65 ± 0.289; *p* = 0.0035), a higher all-cause mortality rate (*p* = 0.005), and a significantly higher COVID-related mortality rate (*p* = 0.00001).

Overall mortality was 1.5-times higher in non-vaccinated patients. Among COVID-19-infected patients, prior vaccination correlated with a 1.3-fold reduction in all-cause mortality. In patients without a history of SARS-CoV-2 infection, vaccination correlated with an even greater reduction in mortality (1.9-fold) (Table 11).

Previous logistic regression analysis confirms that vaccination was associated with a reduction in COVID-induced mortality (OR = 0.041, *p* 0.003, 95% CI 0.05–0.338). Vaccinated patients had about 96% lower odds of COVID-related death compared to unvaccinated patients, but it did not significantly affect overall and cardiovascular mortality.

We assessed the statistical power of the study for the overall mortality parameter. The effect size was calculated for continuous variables, resulting in a mean value of 0.298, although the sample size differs between the deceased and survivor groups, 113 vs. 98, the smaller value, corresponding to the number of survivors, was used in the power calculation. Based on this, a statistical power of 0.583 was obtained, assuming a two tailed test and a maximum Type I error rate of 0.05. The Cramer’s V for categorical variables ranged between 0.05 and 0.242, reflecting varying statistical power across different parameters.

## 4. Discussion

Most of the available evidence regarding the impact of COVID-19 in patients undergoing hemodialysis has been short-term, conducted either during or immediately after the pandemic [20,21,22,23,24,25,26,27,28,29,30,31,32,33,34]. Existing meta-analyses have primarily focused on identifying risk factors for intra-pandemic mortality, characterizing the clinical presentation of the disease in hemodialysis patients, highlighting laboratory predictors of increased intra-pandemic mortality, or assessing short-term post-infectious survival [35,36,37,38,39,40]. However, the long-term consequences of the disease and their impact on patient survival have been less extensively investigated.

COVID-19 infection increased mortality not only through its direct and immediate effects but also via long-term systemic sequelae and the decompensation of patients’ pre-existing latent imbalances. Post-COVID sequelae are collectively referred to as PASC or Long COVID. This entity encompasses, at the pulmonary level, interstitial lung disease and persistent respiratory dysfunction lasting for months or even years after infection [41,42].

Post-COVID cardiac involvement includes arrhythmias (sinus tachycardia or bradycardia, atrial fibrillation or flutter, ventricular tachyarrhythmias), myocarditis and pericarditis, acute coronary syndromes in all their clinical forms, thromboembolic disease, cardiomyopathies, and, in extreme cases, heart failure and sudden cardiac death [43]. Long COVID also comprises a wide spectrum of neuropsychiatric signs and symptoms, ranging from asthenia, fatigue, and insomnia to memory and behavioral disturbances, dizziness, and “brain fog” [44].

This study was designed to assess the long-term impact of COVID-19 infection. We, therefore, considered the 5-year interval (2020–2024)—a period that encompasses the potential manifestation of long COVID in all included patients—as appropriate for the study’s objectives.

Patients undergoing hemodialysis represent a particularly vulnerable population, characterized by multiple comorbidities, for whom COVID-19 infection posed a major challenge. Mortality in this group may occur both in the setting of acute infection—through acute respiratory distress syndrome (ARDS) or secondary to bacterial infections with fatal outcomes—as well as through post-COVID sequelae affecting vital organs. Moreover, during the pandemic, isolation measures and prolonged disinfection protocols considerably reduced access to timely, high-quality medical care, leading to the worsening of comorbidities that are already highly prevalent in hemodialysis patients.

Against this background, this study aimed to evaluate the long-term impact of COVID-19 infection in hemodialysis patients, with a particular focus on the most relevant outcomes—overall mortality and cause-specific mortality. The study cohort is representative at the population level in terms of age, with a mean age of 65 years, a value consistent with that reported by the United States Renal Data System [45].

In contrast to data reported in the literature, our cohort showed a slight predominance of male patients [46]. Our investigation revealed that advanced age and male gender, were substantially correlated with an elevated risk of in-hospital death from COVID-19, consistent with data reported in the literature [13]. While the exact mechanisms underlying the higher mortality rate in male patients remain unclear, it is well established that men have a greater predisposition to cytokine storm and immune-mediated tissue damage compared to women. One hypothesis suggests that this disparity may be attributed to immunomodulatory effects induced by male sex hormones or the expression of X-linked genes. However, studies have shown that in certain countries with higher living standards and advanced medical care, sex-related differences in COVID-19 mortality tend to diminish [47]. It is well established that dialysis vintage is associated with an increased risk of mortality. Specifically, the risk of death rises by approximately 6% for each additional year of dialysis treatment [48].

Chronic inflammation is a frequent complication in hemodialysis patients and contributes to protein-energy wasting (PEW) and malnutrition-inflammation complex syndrome (MICS), cardiovascular disease, erythropoietin resistance, and increased hospitalization and mortality. It is driven by factors such as dialysis membrane bio-incompatibility, endotoxin exposure, vascular access infections, accumulation of uremic toxins, and comorbidities like diabetes, atherosclerosis, and chronic infections [6,7,8,9]. Inflammation may act as a confounding factor in the assessment of mortality among hemodialysis patients. To mitigate inflammation in our cohort, we employed biocompatible dialysis membranes, high-flux hemodialysis or hemodiafiltration, strict infection control protocols, and optimization of dialysis adequacy. The most commonly used inflammatory biomarkers are C-reactive protein (CRP), interleukin-6 (IL-6), ferritin, and tumor necrosis factor-alpha (TNF-α). Although CRP is a well-established biomarker of inflammation, we chose to interpret its value with caution in this study. This decision was due to the heterogeneity of the patient cohorts and the fact that, for patients who died due to COVID-19, we included the average CRP level recorded at the time of hospitalization for the infection. This approach could introduce a significant discrepancy between COVID-positive and COVID-negative patients. Nevertheless, CRP proved to be highly relevant in cases of COVID-related mortality as well as for those with cardiovascular disease-related mortality, and we have presented it accordingly.

Malnutrition represents another significant complication in patients undergoing hemodialysis (HD), with numerous long-term clinical implications. It arises from multiple interrelated causes and plays a major role in worsening patient outcomes including increased morbidity, poor quality of life, and higher mortality rates in this population. Key contributing factors include: anorexia, dietary restrictions, gastrointestinal symptoms, chronic inflammation, inadequate dialysis (uremic toxins buildup), depression, and social isolation. Malnutrition in HD patients is a dynamic and complex condition that requires early identification and multidisciplinary management. Its close association with inflammation and other comorbidities makes it a critical target for improving clinical outcomes and survival in the dialysis population.

The delivered dose of HD therapy (kT/V) is a significant predictor of mortality in these patients. The optimal HD session is influenced by the dialysis duration, type of dialysis, dialyzer permeability, and the quality of vascular access [49]. It is also proven that suboptimal HD sessions adversely influence patient survival, while more intensive dialysis, especially beyond a specific threshold, may enhance survival outcomes. The KDOQI clinical practice guidelines for hemodialysis adequacy recommend a dialysis dose of 4–5 h, 3–4 times per week [50]. During the pandemic, inadequate HD sessions were more frequently observed in elderly patients, whose dialysis parameters could not be fully optimized due to the burden of the present comorbidities. Due to the requirement for performing isolated HD sessions for COVID-19 patients while ensuring the same healthcare workers to serve both COVID-19-positive and COVID-19-negative patients, HD session durations were sometimes reduced, leading to higher ultrafiltration rates/hour and lower blood flow, which increased the incidence of intradialytic complications. Additionally, concerns about SARS-CoV-2 exposure in the community contributed to decreased patient adherence to HD. Moreover, in critical situations, timely interventions for vascular access management were not consistently provided to hemodialysis patients [51].

In our cohort, vascular access for dialysis was predominantly represented by AVF, while the main laboratory data assessed—including hemoglobin, Kt/V, serum creatinine, calcium, phosphorus, PTH, and nutritional markers (albumin, total cholesterol)—were generally within the ranges considered acceptable for this patient population. Across the entire cohort, the risk factors identified for increased mortality included older age, dialysis adequacy (Kt/V), diabetes mellitus (present in 30% of patients), arterial hypertension, overweight status, and low rates of SARS-CoV-2 immunization.

COVID-19 infection predominantly affected older patients, those who were overweight with higher BMI compared to non-COVID individuals, patients with vascular nephropathy, and those with lower rates of SARS-CoV-2 immunization. In this study, no direct correlation was observed between COVID-19 incidence and dialysis vintage, dialysis adequacy, anemia status, mineral-bone metabolism parameters, or nutritional status indicators.

### 4.1. Post-COVID Overall Mortality

Globally, COVID-19 has been associated with mortality rates ranging from 1.4% to 8% in the general population. In HD patients, however, mortality rates have been reported to be up to four times higher [52]. A comparative analysis of deaths recorded over a four-year period in our HD cohort—spanning both the pandemic and post-pandemic periods—revealed notable patterns. COVID-19-positive non-survivors were characterized by older age, shorter dialysis vintage, elevated phosphate, and increased inflammatory markers, with no significant differences in sex distribution, biometric measures, dialysis adequacy, or other laboratory results. According to our analysis, each additional year in HD was associated with a 15% reduction in the odds of COVID-induced death. The possible explanation relates to survivor bias (e.g., patients who have survived longer in HD may be more resilient, while frailer patients died earlier). A distinguishing factor was the higher prevalence of coexisting diabetes mellitus in this subgroup, although without statistical significance.

In our cohort, five-year overall mortality was 53.55%. As expected, it primarily affected elderly patients, those with diabetes, associated malignancies, and significant vascular disease. Deceased patients presented with more pronounced anemia, reduced dialysis adequacy (significantly reduced kT/V, *p* = 0.003), and an imbalanced nutritional status, characterized by being overweight together with comparatively lower serum albumin levels. Although elevated phosphate levels are a well-established predictor of mortality in HD, no statistically significant association was observed between phosphate level and mortality in our cohort. This finding is consistent with the relatively well-controlled levels of iPTH, which were similar in both survivors and deceased patients. However, only anemia and BMI have relevance in logistic regression analysis. Although patients who survived had a higher degree of obesity, we demonstrated the presence of confounding factors in the interpretation of this finding. Specifically, patients with a BMI above 25.19 were younger, had a shorter duration on hemodialysis, a lower degree of anemia, and lower parathyroid hormone (PTH) levels. However, they also had a lower Kt/V and a slightly more pronounced inflammatory profile, highlighting the complexity of the hemodialysis patient, particularly in the context of clinical research and study design. Vaccination was associated with a beneficial effect, contributing to a reduction in long-term all-cause mortality among HD patients.

### 4.2. COVID-Related Mortality

Numerous studies have demonstrated that, owing to an impaired immune response, most patients undergoing hemodialysis develop mild or moderate forms of COVID-19 [53]. However, a severe and progressive course of COVID-19 with poor prognosis has been associated not only with complex underlying diseases but also with patient age and sex [54,55,56]. Patients who developed severe forms of COVID-19 were unvaccinated and had higher BMI values than those with mild or moderate disease. In severe cases, the direct cause of death was the COVID-19 infection itself, whereas in mild and moderate cases, mortality resulted from associated cardiovascular pathology.

COVID-related mortality was recorded in approximately one-fifth of patients infected with SARS-CoV-2. Deaths occurred predominantly during the first pandemic wave, when patients had not yet benefited from specific immunization, with most fatalities being in-hospital and related to severe forms of the disease or decompensation of associated comorbidities. In contrast, during the Omicron wave—when vaccination was available and clinical knowledge regarding disease course, prognosis, and treatment had significantly improved—COVID-related deaths became exceedingly rare.

Patients who died from COVID-19 were typically elderly, unvaccinated, with severe vascular disease, overweight but with a significantly lower BMI compared to COVID survivors, and presented with significantly reduced serum albumin and cholesterol levels. Notably, no statistically significant differences were observed in mortality between patients who did and did not receive antiviral therapy for COVID-19. These findings highlight the crucial role of vaccination and metabolic-nutritional status in shaping COVID-related outcomes among HD patients.

### 4.3. Cardiovascular Mortality

The leading cause of death in both COVID-positive and COVID-negative patients was cardiovascular disease. Cardiovascular mortality affected 24.83% of COVID-positive patients and 39.4% of COVID-negative patients (*p* = 0.006). These findings align with large registry data in HD populations, where cardiovascular disease consistently accounts for approximately 40% of deaths, while in COVID-infected cohorts respiratory causes often predominate and reduce the relative proportion of cardiovascular deaths [57,58].

Even if multiple predictors of cardiovascular mortality were identified in linear regression, multivariate logistic analysis revealed no notable differences regarding gender, dialysis vintage, vascular access, comorbidities including diabetes mellitus, except for hemoglobin and kt/V. This finding is consistent with pre-COVID HD published data, in which lower hemoglobin levels independently predict all-cause and cardiovascular mortality, and higher dialysis adequacy (Kt/V) is associated with improved survival and reduced cardiovascular risk [58,59]. Among COVID-positive HD patients, one recent analysis found that off-target single-pool Kt/V was associated with increased 30-day mortality, suggesting dialysis adequacy may remain important in this context, although hemoglobin has not yet been confirmed as an independent predictor in COVID-specific cohorts [54]. In this study a previous documented cardiovascular disease relates with 6-fold increasing risk of cardiac death.

Deaths due to infections and malignancies were each recorded in fewer than 5% of patients, with slightly higher incidence among COVID-negative individuals.

In our cohort, the only parameter that differed significantly was the vaccination rate, which was higher among COVID-negative patients, who also exhibited lower overall mortality. Comparable findings have been reported in other hemodialysis cohorts. Tu et al. showed that vaccinated patients had significantly lower mortality (6.3% vs. 14.4%, *p* = 0.049) and fewer cardiac complications after COVID-19 infection [60]. In contrast, Okamoto et al. observed no long-term increase in cardiovascular events or mortality after mild COVID-19 in the post-Omicron era, suggesting that infection severity and vaccination coverage critically shape outcomes in this population [61].

This apparent paradox—higher cardiovascular mortality among COVID-negative patients despite their lower overall mortality—may highlight the complex interplay between immunization status, nutritional markers such as serum albumin, and cause-specific outcomes in HD population.

### 4.4. Impact of COVID-19 Vaccination

COVID-19 vaccination among hemodialysis patients was achieved at variable rates worldwide, and the efficacy of immunization has represented a central topic of numerous studies. The main vaccine technologies assessed comprise inactivated virus vaccines, non-replicating viral vector vaccines, RNA- and DNA-based vaccines, protein subunit vaccines, and vaccines utilizing virus-like particles (VLPs) [62]. Barriers to vaccination in hemodialysis patients are well-documented and multifactorial, spanning patient-level (lack of awareness, fear of side effects or mistrust in vaccines, cognitive impairment, cultural barriers), provider-level, and system-level factors (lack of protocols and registries). Initial concerns regarding the safety of vaccination in HD patients stemmed mainly from reports of thromboembolic events in the general population [63]. Consequently, various available vaccines—such as the BNT162b2 mRNA vaccine and the inactivated CoronaVac vaccine—were systematically evaluated in an HD population, with direct comparisons made between them [64,65]. The consistent conclusion across these studies was that COVID-19 vaccination is safe in patients undergoing hemodialysis [62]. Regarding efficacy, although the humoral immune response in HD patients was found to be suboptimal, studies demonstrated that the cellular immune response was sufficiently robust to provide protection against severe forms of COVID-19 [19]. To improve the antibody-mediated response, it was recommended that the anti-spike antibody be monitored and booster doses administered as needed [66]. The protective effects of vaccination on cardiovascular pathology are due to the prevention of severe endothelial disfunction, hypercoagulability, cytokine storm, and hypoxia associated with COVID infection. The lack of COVID-19 vaccination in HD patients, given the high prevalence of atherosclerosis, left ventricular hypertrophy, diabetes, hypertension, and chronic inflammation, may significantly increase the risk of cardiovascular mortality. In our cohort, the short-term clinical impact of vaccination was substantial. Notably, most patients who died during COVID-19-related hospitalization were unvaccinated. Overall, vaccine acceptance was low, 38.38%, influenced by the general mistrust surrounding vaccination during the pandemic. Patients who refused immunization had significantly lower adherence to dialysis sessions (Kt/V 1.53 ± 0.322 vs. 1.65 ± 0.289, *p* = 0.0035). Although antiviral treatment was more frequently administered in the unvaccinated group, mortality was significantly higher among them with SARS-CoV-2 infection representing the primary cause of death, in line with findings reported in the literature.

The long-term effects of COVID-19 vaccination were manifold. Vaccination significantly reduced COVID-related mortality, while its influence on overall mortality was more modest. Among patients who experienced COVID-19 infection, vaccination lowered the overall mortality rate by 1.3-fold (from 60.82% to 46.15%), whereas in COVID-negative patients, vaccination reduced mortality by 1.9-fold (from 63.63% to 33.33%).

Post-vaccination serological monitoring—particularly the measurement of anti-SARS-CoV-2 spike protein antibodies—has emerged as a valuable tool in assessing vaccine-induced immunity in this population. Studies have shown that seroconversion rates in HD patients are lower compared to the general population, and antibody titers tend to wane more rapidly over time [67]. Factors influencing the immune response include age, dialysis vintage, comorbidities (e.g., diabetes), nutritional and inflammatory status, and immunosuppressive therapy. Repeated booster doses have been shown to improve seroconversion and antibody persistence in HD patients, though variability remains. Serological monitoring can help identify non-responders or low responders who may benefit from additional vaccine doses or alternative prophylactic strategies, such as monoclonal antibodies. Given the vulnerability of the HD population, integrating routine post-vaccination antibody testing might help optimize protection strategies against COVID-19, though standardized protocols for testing and interpretation are still under development.

The strength of this study lies in its extended timeframe, spanning both the pandemic and post-pandemic periods, its conduct in one of the largest hospitals in Western Romania designated for the treatment of COVID-19 patients, and the comprehensive inclusion of multivariate analyses that addressed the full spectrum of parameters specific to hemodialysis patients—demographic and biometric data, comorbidities, dialysis adequacy indices, and laboratory markers relevant to the hemodialysis population. Consequently, the statistical robustness of the findings is considerably enhanced. The intra- and post-pandemic mortality model developed here may be extrapolated and used as a predictive tool in future pandemic scenarios, while the data obtained can be integrated into meta-analyses with greater statistical power. Importantly, vaccination in hemodialysis patients demonstrated an overwhelming benefit, potentially halving all-cause mortality in similar epidemiological contexts.

Study limitations include its single-center design, which inherently restricts the number of patients included, and the inclusion of a racially homogeneous patient population, factors which restrict the generalizability of the proposed nomogram model. In addition, mortality was not stratified according to the specific SARS-CoV-2 variant responsible for infection and the vaccination efficacy was not evaluated. However, vaccine efficacy was not evaluated, partly because national guidelines did not include the administration of booster doses for hemodialysis patients and partly based on the assumption that the standard three-dose regimen would provide a sufficient antibody response. The lack of a control group constitutes another study limitation. For the evaluation of mortality and vaccine efficacy, the control groups were selected from the hemodialysis population during the study period. However, comparing overall and cardiovascular mortality before versus after the pandemic would require extending the evaluation period to more than 10 years, during which significant changes occurred both in the patient population and in the clinical practice guidelines used in their management. Future multicenter or population-level studies are needed to confirm our findings in other settings. Nationwide data collection would represent a significant step forward in achieving a deeper understanding of the clinical behavior and outcomes of this vulnerable population in the context of pandemic scenarios.

## 5. Conclusions

Patients undergoing hemodialysis represent a vulnerable population with elevated mortality during the COVID-19 pandemic. Older age, overweight, associated vascular disease, anemia, proinflammatory status, underlying cardiovascular pathology, and lack of vaccination emerged as the main risk factors for mortality among patients who experienced COVID-19 infection. Cardiovascular disease was the leading cause of death throughout the study period, irrespective of prior infection, with mortality predominantly driven by the older age, overweight, low dialysis efficacy, and cardiovascular pathology. COVID-related deaths occurred almost exclusively in unvaccinated patients, primarily during the first wave of the pandemic. Vaccination reduced COVID mortality risk by 43-fold across the study period, while overall mortality remained uninfected.

## Figures and Tables

**Table 1:** Baseline characteristics of the study cohort; comparative evaluation of COVID-19-Positive and -Negative Patients.

Parameters M/SD		All Patients (*n* = 211)	COVID (+) (*n* = 149)	COVID (-) (*n* = 62)	*p*-Value
Age		65.659 ± 13.299	66.86 ± 11.801	62.774 ± 16.092	*0.021*
Gender (male)		118 (55.92%)	86 (57.71%)	34 (54.83%)	0.816
HD vintage		6.924 ± 5.482	7.02 ± 5.566	6.69 ± 5.311	0.347
AVF		139 (65.87%)	101 (67.78%)	38 (61.29%)	0.455
Previous Tx		17 (8.05%)	11 (7.38%)	6 (9.69%)	0.779
Diabetes mellitus		66 (31.27%)	32 (21.47%)	18 (29.03)	0.318
AHT		192 (90.99%)	136 (91.27%)	56 (90.32%)	0.965
Neoplastic diseases		33 (15.63%)	24 (16.10%)	9 (14.51%)	0.934
Underlying Kidney disease	CGN	85 (40.28%)	51 (34.22%)	34 (54.83%)	*0.0277*
	VN	44 (20.85%)	37 (24.83%)	7 (11.29%)	*0.043*
	DN	41 (19.43%)	32 (21.47%)	9 (14.51%)	0.330
	PKD	13 (6.16%)	11 (22.44%)	2 (3.22%)	0.406
BMI		25.806 ± 6.192	26.52 ± 5.949	24.32 ± 6.462	*0.009*
Hemoglobin g/dL		10.702 ± 1.499	10.67 ± 1.518	10.78 ± 1.471	0.500
Kt/V		1.530 ± 0.315	1.57 ± 0.312	1.59 ± 0.323	0.360
Serum creatinine mg/dL		7.691 ± 2.427	8.66 ± 12.303	7.62 ± 2.332	0.258
Serum calcium mg/dL		8.91 ± 0.949	8.92 ± 0.963	8.88 ± 0.922	0.390
Serum phosphate mg/dL		5.09 ± 1.848	5.12 ± 1.872	5 ± 1.803	0.326
iPTH pg/mL		4.9	348.29 ± 341.42	432.21 ± 577.15	0.102
Cholesterol mg/dL		152.47 ± 39.257	152.97 ± 39.649	151.23 ± 39.066	0.386
Albumin g/dL		149	10.67 ± 1.518	3.71 ± 0.370	0.408
Serum bicarbonate mmol/L			1.57 ± 0.312	24.28 ± 3.578	0.251
COVID vaccination		81 (38.38%)	52 (34.89%)	29 (46.77%)	*0.00825*
Death		113 (53.55%)	83 (55.7%)	30 (48.38%)	0.412
Age at death		69.51 ± 11.943	70.27 ± 9.976	68.67 ± 15.379	0.299
COVID induced mortality		34 (16.11%)	34 (22.81%)	0	*0.000*
Cardiovascular mortality		59 (27.96%)	37 (24.83%)	22 (39.4%)	*0.006*
Infectious mortality		7 (3.31%)	4 (2.68%)	3 (4.83%)	0.570
Neoplastic mortality		10 (4.73%)	7 (4.69%)	3 (4.83%)	0.907

Abbreviations: M = mean, SD = standard deviation, HD = hemodialysis; Tx = transplantation; AVF = arteriovenous fistula, AHT = arterial hypertension; BMI = body mass index; CGN = chronic glomerulonephritis; VN = vascular nephropathy; DN = diabetic nephropathy; PKD = polycystic kidney disease; iPTH = intact parathormone; italics = statistically significant.

**Table 2:** Clinical and demographic characteristics of deceased patients and survivors during follow-up.

Parameter (M ± SD) no/%		Death Patients (*n* = 113) 53.55%	Survivors (*n* = 98) 46.44%	*p*-Value
Age		69.51 ± 11.943	61.21 ± 13.451	*0.00001*
Gender (male)		67 (58.29%)	51 (52.04%)	0.358
HD vintage		6.99 ± 6.011	6.85 ± 4.836	0.424
AVF		69 (58.29%)	70 (71.42%)	0.150
Previous Tx		7 (6.19%)	10 (10.2%)	0.415
Diabetes mellitus		45 (39.82%)	21 (21.42%)	*0.006*
AHT		100 (88.49%)	92 (93.87%)	0.262
Neoplastic diseases		24 (21.23%)	9 (9.18%)	0.057
Underlying Kidney disease	CGN	*34 (30.03%)*	51 (52.04%)	*0.001*
	VN	*32 (30.03%)*	12 (12.24%)	*0.007*
	DN	*24 (21.23%)*	17 (17.34%)	0.059
	PKD	*7 (6.19%)*	6 (6.12%)	0.790
BMI		*25.19 ± 6.572*	26.69 ± 6.602	*0.040*
Hemoglobin g/dL		*10.32 ± 1.630*	*11.16 ± 1.195*	*0.00002*
kT/v		*1.52 ± 0.347*	1.63 ± 0.264	*0.003*
Creatinine mg/dL		7.22 ± 2.399	9.67 ± 15.05	*0.045*
Calcium mg/dL		8.83 ± 1.004	9 ± 0.874	0.091
Phosphate mg/dL		5.22 ± 2.006	4.92 ± 1.629	0.119
iPTH pg/mL		379.72 ± 390.46	365.26 ± 468.183	0.406
Cholesterol mg/dL		149.19 ± 39.21	156.19 ± 39.47	0.101
Albumin g/dL		*3.65 ± 0.566*	*3.83 ± 0.332*	*0.0036*
COVID vaccination		*33 (29.20%)*	48 (48.97%)	*0.0032*
COVID+		*73 (64.60%)*	66 (67.34%)	0.784
Antiviral treatment (hospitalised patients) lopinavir + ritonavir favipiravir remdesivir molnupiravir darunavir		27 (23.89%) 13 6 7 - 1	19 (19.38%) 8 3 1 2 5	0.532

Abbreviations: M = mean, SD = standard deviation, HD = hemodialysis; Tx = transplantation; AVF = arteriovenous fistula, AHT = arterial hypertension; BMI = body mass index; CGN = chronic glomerulonephritis; VN = vascular nephropathy; DN = diabetic nephropathy; PKD = polycystic kidney disease; iPTH = intact parathormone; italics = statistically significant.

**Table 3:** Overall mortality model coefficients.

Parameter	OR	*p*	95% Confidence Interval	
			Lower	Upper
Age	1.078	<0.001	1.038	1.119
BMI	0.868	<0.001	0.803	0.939
Hemoglobin g/dL	0.581	<0.001	0.427	0.791
Calcium mg/dL	1.306	0.240	0.837	2.038
Phosphate mg/dL	1.351	0.025	1.039	1.758
iPTH pg/mL	0.999	0.267	0.998	1.000
Kt/v	0.138	0.013	0.029	0.658
Hs-CRP mg/L	1.014	<0.001	1.006	1.023
DM	1.982	0.143	0.793	4.952
Ischemic cardiopathy	3.058	0.009	1.317	7.101
Neoplastic diseases	2.820	0.067	0.932	8.534
AntiCOVID-19 vaccination	0.552	0.156	0.243	1.253
Anti-SARS-CoV2 treatment	0.345	0.054	0.117	1.020

**Table 4:** Mortality in COVID-19-positive versus COVID-19-negative patients.

Parameter	General Mortality				Cardiovascular Mortality			
	All Patients (*n* = 211)	COVID (+) (*n* = 149)	COVID (-) (*n* = 62)	*p*-Value	All Patients (*n* = 211)	COVID (+) (*n* = 149)	COVID (-) (*n* = 62)	*p*-Value
Death	113 53.55%	83 55.7%	30 48.38%	0.332	59 27.96%	37 24.83%	22 35.48%	*p* 0.160
Age	69.51 ± 11.943	70.27 ± 9.976	69.43	0.133	70.72 ± 11.76	70.56 ± 9.723	70.14 ± 14.376	0.447
Gender (male)	67	51 (61.44%)	16 (53.33%)	0.438	27 (45.76%)	16 (43.24%)	11 (50%)	0.888
HD vintage	6.99 ± 6.011	6.71 ± 5.909	7.77	0.205	8.79 ± 6.69	8.63 ± 6.817	9.09 ± 6.81	0.402
AVF	69.51 ± 11.943	54 (65.06%)	15 (50%)	0.147	35 (59.32%)	24 (66.66%)	11 (50%)	0.325
Previous Tx	7	5 (6.02%)	2 (6.66%)	0.9	2 (3.38%)	2 (5.4%)	0	
DM	45	33 (39.75%)	12 (40%)	0.655	22 (37.28%)	13 (36.11%)	9 (40.90%)	0.931
AHT	100	73 (87.95%)	27 (90%)	0.466	56 (94.91%)	34 (99.44%)	21 (95.45%)	0.658
Neoplastic diseases	24	18 (21.68%)	6 (20%)	0.420	7 (11.86%)	5 (13.51%)	2 (9.09%)	
BMI	25.19 ± 6.572	25.45 ± 6.316	24.42	0.233	24.35 ± 6.77	24.99 ± 6.303	23.49 ± 7.635	0.212
Hb	10.32 ± 1.630	10.32 ± 1.588	10.31	0.48	10.43 ± 1.55	10.59 ± 1.482	10.18 ± 1.703	0.168
kT/v	1.52 ± 0.347	1.62 ± 0.254	1.48	0.274	1.53 ± 0.33	1.56 ± 0.345	1.49 ± 0.331	0.239
Creatinine	7.22 ± 2.399	7.3 ± 2.284	7.02	0.297	6.29 ± 2.28	7.268 ± 2.054	6.763 ± 2.464	0.202
Calcium	8.83 ± 1.004	8.78 ± 1.069	8.96	0.203	9.00 ± 0.79	8.978 ± 0.862	9.059 ± 0.712	0.357
Phosphate	5.22 ± 2.01	5.29 ± 2.049	5.03	0.270	5.07 ± 1.76	5.339 ± 1.824	4.739 ± 1.593	0.104
iPTH	379.72 ± 390.46	357.77 ± 336.96	441.03	0.163	476.32 ± 459.05	464.36 ± 391.4	506.09 ± 568.36	0.370
Cholesterol	149.19 ± 39.21	146.54 ± 37.669	156.56	0.119	148.22 ± 37.76	147.77 ± 34.84	149.71 ± 43.75	0.427
Albumin	3.65 ± 0.566	3.66 ± 0.611	3.62	0.367	3.73 ± 0.42	3.8 ± 0.429	3.6 ± 0.415	*0.049*
Anti SARS COV2 vaccination	33 (29.20%)	24 (28.91%)	9 (33.3%)	0.611	28 (42.42%)	17 (47.22%)	5 (22.72%)	0.062

Abbreviations: HD = hemodialysis; Tx = transplantation; DM = diabetes mellitus; AHT = arterial hypertension; BMI = body mass index; iPTH = intact parathormone; italics = statistically significant.

**Table 5:** Comparative analysis of survivors and non-survivors among COVID-19-positive patients.

Patients’ Characteristics		COVID (+) Death (*n* = 83)	COVID Survivors (*n* = 66)	*p*-Value
Age		70.27 ± 9.976	62.58 ± 12.574	*0.000027*
Gender (male)		51 (61.44)	33 (50%)	0.217
HD vintage		6.71 ± 5.909	7.41 ± 5.126	0.223
Arteriovenous fistula		54 (65.06%)	47	0.534
Previous Tx		5 (6.02%)	6 (9.09%)	0.692
Diabetes mellitus		33 (39.75%)	15 (22.72%)	*0.042*
AHT		73 (87.95%)	63 (95.45%)	0.186
Neoplastic diseases		18 (21.68%)	6 (9.09%)	0.063
Underlying Kidney disease	CGN	19 (22.89%)	32 (48.48)	*0.001*
	VN	28 (33.73%)	9 (13.63)	*0.008*
	DN	20 (24.09%)	12 (18.18)	0.501
	PKD	5 (6.02%)	6 (9.09%)	0.692
BMI		25.45 ± 6.316	27.87 ± 5.193	*0.006*
Hemoglobin g/dL		10.32 ± 1.588	11.13 ± 1.301	*0.0005*
kT/v		1.62 ± 0.254	1.53 ± 0.348	*0.040*
Creatinine mg/dL		7.3 ± 2.284	10.36 ± 18.842	0.065
Calcium mg/dL		8.78 ± 1.069	9.1 ± 0.774	*0.022*
Phosphate mg/dL		5.29 ± 2.049	4.9 ± 1.592	0.103
iPTH pg/mL		357.77 ± 336.96	335.05 ± 350.08	0.350
Cholesterol mg/dL		146.54 ± 37.669	160.86 ± 40.864	*0.014*
Albumin g/dL		*3.66 ± 0.611*	*3.87 ± 0.356*	*0.020*
Anti COVID-19 vaccination		24 (28.91%)	28 (42.42%)	0.122

Abbreviations: HD = hemodialysis; Tx = transplantation; AHT = arterial hypertension; BMI = body mass index; CGN = chronic glomerulonephritis; VN = vascular nephropathy; DN = diabetic nephropathy; PKD = polycystic kidney disease; iPTH = intact parathormone; italics = statistically significant.

**Table 6:** Mortality in COVID-19-positive patients model coefficients.

Parameter	OR	*p*	95% Confidence Interval	
			Lower	Upper
Age	1.069	0.002	1.025	1.116
BMI	0.885	0.009	0.808	0.970
Hemoglobin g/dL	0.641	0.014	0.449	0.915
Albumin g/dL	1.590	0.407	0.534	4.751
Cholesterol mg/dL	0.990	0.117	0.978	1.003
Kt/v	0.493	0.422	0.880	2.768
Hs-CRP mg/L	1.015	<0.001	1.006	1.024
Diabetic nephropathy	1.591	0.450	0.477	5.307
Ischemic cardiopathy	5.683	<0.001	2.160	14.952
Neoplastic diseases	3.489	0.086	0.840	14.497
Anti-COVID-19 vaccination	0.700	0.475	0.264	1.861
Anti-SARS-CoV2 treatment	0.539	0.282	0.175	1.659

**Table 7:** Comparative analysis between COVID-19-induced deaths and survivors.

Patients’ Characteristics		COVID Induced Death (*n* = 34)	COVID (+) Survivors (*n* = 66)	*p*-Value
Age		*69.53 ± 11.38*	*62.58 ± 12.574*	*0.004*
Gender (male)		23 (67.64%)	33 (50%)	0.092
HD vintage		5.09 ± 4.69	7.41 ± 5.126	0.014
Arteriovenous fistula		21 (61.76%)	47	0.53
Previous Tx		2 (5.88%)	6 (9.09%)	0.69
Diabetes mellitus		12 (35.29%)	15 (22.72%)	0.269
Arterial hypertension		27 (79.41%)	63 (95.45%)	0.063
Neoplastic diseases		8 (23.52%)	6 (9.09%)	0.082
Underlying Kidney disease	CGN	5 (14.70%)	32 (48.48)	*0.001*
	VN	13 (38.23%)	9 (13.63)	*0.008*
	DN	12 (35.29%)	12 (18.18)	0.089
	PKD	2 (5.88%)	6 (9.09%)	0.53
BMI		26.68 ± 6.4	27.87 ± 5.193	0.160
Hemoglobin g/dL		10.3 ± 1.70	11.13 ± 1.301	0.004
kT/v		1.51 ± 0.35	1.53 ± 0.348	*0.036*
Creatinine mg/dL		7.44 ± 2.64	10.36 ± 18.842	0.178
Calcium mg/dL		8.66 ± 1	9.1 ± 0.774	0.5
Phosphate mg/dL		5.64 ± 2.39	4.9 ± 1.592	*0.035*
iPTH pg/mL		295.15 ± 283.55	335.05 ± 350.08	0.286
Cholesterol mg/dL		153.68 ± 40.76	160.86 ± 40.864	0.5
Albumin g/dL		3.33 ± 0.77	3.87 ± 0.356	0.007
CRP mg/L		149.75 ± 116.54	36.71 ± 54.43	<0.001
COVID vaccination		1 (2.94%)	28 (42.42%)	<0.0001
Antiviral treatment		17 (50%)	19 (28.78%)	0.060

Abbreviations: HD = hemodialysis; Tx = transplantation; AHT = arterial hypertension; BMI = body mass index; CGN = chronic glomerulonephritis; VN = vascular nephropathy; DN = diabetic nephropathy; PKD = polycystic kidney disease; iPTH = intact parathormone; italics = statistically significant.

**Table 8:** Multiple logistic regression- COVID-19-related mortality model coefficients.

Parameter	OR	*p*	95% Confidence Interval	
			Lower	Upper
Age	1.039	0.114	0.991	1.091
HD vintage	0.854	0.013	0.753	0.967
Albumin g/dL	0.581	0.255	0.228	1.481
Phosphate mg/dL	1.341	0.044	1.007	1.784
Hs-CRP mg/L	1.012	<0.001	1.006	1.019
Anti-COVID-19 vaccination	0.041	0.003	0.005	0.338
Anti-SARS-CoV2 treatment	1.921	0.242	0.644	5.727

**Table 9:** Comparison of survivors and non-survivors due to cardiovascular causes.

Patients’ Characteristics		Cardiovascular Death *n* = 59	Survivors (*n* = 98)	*p*-Value
Age		70.72 ± 11.76	61.21 ± 13.451	0.0002
Gender (male)		27 (45.76%)	51 (52.04%)	0.55
Hemodialysis vintage		8.79 ± 6.69	6.85 ± 4.836	0.0001
Arteriovenous fistula		35 (59.32%)	70 (71.42%)	0.16
Diabetes mellitus		22 (37.28%)	21 (21.42%)	0.048
Arterial hypertension		56 (94.91%)	92 (93.87%)	0.933
Neoplastic diseases		7 (11.86%)	9 (9.18%)	0.95
Underlying kidney disease	CGN	23 (38.98%)	62 (63.26%)	0.005
	VN	16 (27.11%)	28 (28.57%)	0.98
	DN	9 (15.25%)	32 (32.65%)	0.026
	PKD	5 (8.47%)	8 (8.16%)	0.81
BMI		24.35 ± 6.77	26.69 ± 6.602	0.013
Hemoglobin g/dL		10.43 ± 1.55	11.16 ± 1.195	0.0007
Hs-CRP mg/L		54.136 ± 61.764	29.790 ± 47.254	0.007
kT/v		1.53 ± 0.33	1.63 ± 0.264	0.023
Creatinine mg/dL		6.29 ± 2.28	9.67 ± 15.05	0.5
Calcium mg/dL		9.00 ± 0.79	9 ± 0.874	0.49
Phosphate mg/dL		5.07 ± 1.76	4.92 ± 1.629	0.29
iPTH pg/mL		476.32 ± 459.05	365.26 ± 468.183	0.078
Cholesterol mg/dL		148.22 ± 37.76	156.19 ± 39.47	0.11
Albumin g/dL		3.73 ± 0.42	3.83 ± 0.332	0.051
COVID-19 vaccination		22 (37.28%)	48 (48.97%)	0.20
COVID+		37 (62.71%)	66 (67.34%)	0.67

Abbreviations: BMI = body mass index; CGN = chronic glomerulonephritis; VN = vascular nephropathy; DN = diabetic nephropathy; PKD = polycystic kidney disease; iPTH = intact parathormone.

**Table 10:** Multivariable logistic regression analysis of predictors of cardiovascular mortality.

Parameter	OR	*p*	95% Confidence Interval	
			Lower	Upper
Age	1.061	0.010	1.014	1.115
HD vintage	1.084	0.064	0.995	1.180
BMI	0.818	<0.001	0.729	0.916
Albumin g/dL	1.381	0.648	0.346	5.513
Hemoglobin g/dL	0.466	<0.001	0.300	0.724
Cholesterol mg/dL	1.003	0.686	0.989	1.016
Calcium mg/dL	1.299	0.367	0.736	2.292
Phosphate mg/dL	1.101	0.549	0.803	1.510
Kt/v	0.068	0.014	0.008	0.583
Hs-CRP mg/L	1.006	0.199	0.997	1.014
DM	2.609	0.08	0.866	7.857
Ischemic cardiopathy	5.837	<0.001	2.053	16.594

**Table 11:** Impact of vaccination on the outcomes of hemodialysis patients.

Parameters (M ± SD); no/%		Vaccine (+) (*n* = 81)	Vaccine (-) (*n* = 130)	*p*-Value
Age		66.37 ± 11.66 69	67.22 ± 14.24 68	0.270
Gender (male)		44 (54.32%)	74 (56.92%)	0.819
HD vintage		6.96 ± 5.294 5	6.9 ± 5.614 5	0.468
AVF		54 (66.66)	85 (65.38%)	0.966
Diabetes mellitus		25 (30.86%)	41 (31.53%)	0.960
Arterial hypertension		74 (91.35%)	118 (90.76%)	0.918
Neoplastic diseases		9 (11.11%)	25 (19.23%)	0.171
Underlying Kidney disease	CGN	30 (37,03%)	55 (42.30%)	0.538
	VN	17 (20.98%)	27 (20.76%)	0.819
	DN	17 (20.98%)	24 (18.46%)	0.785
BMI		26.12 ± 5.837	25.73 ± 6.386	0.329
Hemoglobin g/dL		10.73 ± 1.427	10.69 ± 1.554	0.419
kT/v		1.65 ± 0.289	1.53 ± 0.322	0.0035
Creatinine mg/dL		7.66 ± 2.154	8.79 ± 13.21	0.223
Calcium mg/dL		8.94 ± 0.995	8.89 ± 0.949	0.335
Phosphate mg/dL		5.01 ± 1.656	5.13 ± 1.962	0.317
iPTH pg/mL		343.34 ± 324.71	309.41 ± 474.22	0.227
Albumin g/dL		3.77 ± 0.351	3.71 ± 0.545	0.219
COVID (+)		52 (64.19%)	97 (74.61%)	0.182
Antiviral treatment		14 (17.28%)	32 (24.61%)	0.278
Death		33 (40.74%)	80 (62.53%)	0.005
Age at death		69.4 ± 12.67	70.65 ± 10.158	0.324
Death/COVID		1 (1.23%)	37 (28.46%)	0.00001
Death/cardiovascular		22 (27.16%)	35 (26.92%)	0.964
Death/infections		4 (4.93%)	3 (2.30%)	0.520
Death/neoplastic diseases		5 (6.17%)	5 (3.84%)	0.659

Abbreviations: M = mean, SD = standard deviation, HD = hemodialysis; BMI = body mass index; CGN = chronic glomerulonephritis; VN = vascular nephropathy; DN = diabetic nephropathy; iPTH = intact parathormone.

## Data Availability

The data presented in this study are available on request from the corresponding author. The data are not publicly available due to ethical reasons.
